# Social distancing mediates clinical work and depression: impact of the COVID-19 pandemic on nurses

**DOI:** 10.3389/fpubh.2024.1437766

**Published:** 2024-11-22

**Authors:** Shu-Chen Chen, Tony Szu-Hsien Lee, En Chao

**Affiliations:** ^1^Department of Health Promotion and Health Education, National Taiwan Normal University, Taipei, Taiwan; ^2^Tri-Service General Hospital Songshan Branch, Taipei, Taiwan; ^3^National Defense Medical Center of General Education, Taipei, Taiwan

**Keywords:** COVID-19, depression, nursing, social distancing, tenure, work unit, mediation

## Abstract

**Background:**

The psychological well-being of nurses, during the COVID-19 pandemic has become a critical area of concern. This study examines the psychological impact of the pandemic on nurses, focusing on the mediating role of perceived social distance between work units and depression.

**Methods:**

This study employed a cross-sectional design with respondent driven sampling. Anonymous questionnaire consisting of demographics, perceived social distancing and depression were distributed through email, Facebook, LINE, and other online platforms by key informants of nurses. A total of 1,064 volunteered questionnaires were collected, consisting of 1,032 females and 32 males.

**Results:**

Data showed that 517 (48.9%) nurses may have depression. Results from the structure equation modeling indicated that perception of social distancing mediates between individuals in units of care and depression, while unit and tenure of nursing work were negatively associated with depression.

**Conclusion:**

The study findings support that perceived social distancing due to COVID-19 pandemic from others toward nurses may have influential impact on elevated depression, especially for nurses worked in the acute critical care. Thus, emotional support should be emphasized, and avenues for stress relief should be provided as well as creating a supportive workplace environment is crucial to nurses and patient safety.

## Introduction

In 2003, Severe Acute Respiratory Syndrome (SARS) swept across the globe, with nurses taking on roles in epidemic prevention and safeguarding public health. Since the end of 2019, with the first case of unexplained pneumonia reported in Wuhan, China, the novel coronavirus disease (COVID-19) erupted globally within a short span of about 2 months. Due to insufficient understanding of the virus and its highly infectious nature, and the lack of established protective equipment and standards for healthcare personnel, healthcare workers were infected (1). Therefore, the World Health Organization (WHO) convened an emergency meeting in March 2020 and declared the outbreak of this emerging infectious disease as a global pandemic.

Previous studies identified that the infection rate among healthcare workers in Wuhan, China, during the early stages of the COVID-19 outbreak ranged from 3.5 to 29% (2, 3). In the absence of a vaccine and with a high mortality rate post-infection, isolation of physical contacts and city lockdowns became primary control measures (2, 3). Consequently, nurses once again donned their armor during the pandemic, shuttling between hospitals and homes, facing tests of physical and emotional endurance. As of May 5, 2023, the World Health Organization officially announced the cessation of the global public health emergency. The COVID-19 pandemic has persisted for over 3 years, resulting in over 7.6 billion infections worldwide. As of September 7, 2023, statistics from Taiwan indicate a total of 10,241,523 confirmed cases and 17,668 deaths (4).

In the literature, severe large-scale infectious diseases such as SARS have been studied and results showed that they can induce negative emotions such as worry, depression, and frustration due to the uncertainty and stigma associated with disease (5–7). Besides posing a threat to human physical health, they also impose significant psychological burdens (8). The transmission route of COVID-19 involves close contact, and early prevention and control measures during the initial outbreak involved isolating high-risk individuals. Therefore, individuals with relevant TOCC (Travel, Occupation, Contact, and Cluster history) or respiratory symptoms were required to undergo home quarantine or isolation (9).

The severity of the COVID-19 pandemic not only poses a serious threat to the health of humanity but also significantly increases the workload of clinical healthcare workers. Healthcare professionals working in hospitals serve as frontline guardians in epidemic prevention and consequently, more vulnerable to mental distress and health. Hsieh et al. (10) found that healthcare personnel infected with SARS exhibited coping behaviors during isolation, including seeking self-help, searching for the source of infection, protecting family members, reflecting on their professional stance, and seeking spiritual solace. Chen et al. (11) pointed out that the various pressures and concerns faced by nurses will have an immediate and lasting significant impact on the psychological well-being of nursing staff. Research has indicated that the COVID-19 pandemic has had a significant impact on healthcare workers, increasing the risk of psychological distress among healthcare professionals (12).

For healthcare professionals who worked in critical care unit and came into close contact with potential COVID-19 patients may face much higher risks of infection and psychological burden. Wei et al. (13) investigated the impact of the COVID-19 outbreak on the physical and mental health of healthcare institution staff, workplace fatigue levels, and stress from caring for patients with highly contagious infectious diseases during the early stages of the outbreak. The results revealed that among 224 participants, those who had experienced the 2003 SARS epidemic exhibited significantly higher levels of workplace fatigue and stress from caring for patients with highly contagious infectious diseases compared to those who had not experienced SARS, demonstrating statistically significant differences. A qualitative descriptive study of nurses’ perceptions of work and life under COVID-19 revealed that nurses faced unsafe work environments and the shadow of suffering and death during the COVID-19 pandemic (14). They also experienced a lack of support and understanding from managers and inadequate protective equipment, highlighting the need for better healthcare infrastructure and mental health support.

During the early stages of the COVID-19 pandemic, inadequate information about the emerging infectious disease, coupled with the inability to grasp the virus’s strong infectivity and the failure to establish timely comprehensive protective measures, led to many healthcare workers contracting COVID-19. Frontline healthcare workers diagnosed with or suspected of having COVID-19 were more likely to experience depression, anxiety, and insomnia. Feng et al. (15) pointed out that COVID-19 has extremely strong infectivity. Clinical nurses, as frontline caregivers, are at a higher risk of infection due to their prolonged and frequent close contact with patients, resulting in significant negative emotions when caring for COVID-19 patients, including anxiety, insomnia, and symptoms of post-traumatic stress disorder. They may also face loss of family support, concerns about becoming infected and stigmatized, and reduced interpersonal relationships.

Healthcare workers caring for COVID-19 patients may face social stigma, discrimination, and shame in the community, resulting in experiences such as avoidance, being perceived as unclean, discrimination against family members, and intrusion by others (16). Stigma is the process by which an individual is discredited, linking certain traits with moral norms, thereby causing the person to be stigmatized and devalued by society (17). Examples include individuals with physical deformities, mental illness, substance users, sex workers, or those rejected for other reasons. Such individuals must continually strive to adapt to unstable social identities and may feel offended in terms of self-image. Scholars interpret social stigma or stigmatization as a form of societal devaluation, leading individuals to be marginalized and classified into undesirable, rejected identities. However, biases and stereotypes are common in everyday life and can lead to interpersonal harm. If experienced by healthcare professionals, it can lead to unimaginable consequences (18). Due to social stigma experienced in interpersonal interactions, most nursing personnel are concerned about issues such as housing during the pandemic, fears of social isolation, and worries about infecting loved ones, leading to emotional distress such as anxiety, agitation, and feelings of depression. Coping mechanisms include self-isolation, feelings of guilt, and concealment, highlighting the need for support systems for such nursing personnel (15, 19).

In summary, previous studies have documented that increased depression in nurses is associated with direct patient care (13), stigma (16), and tenure (11, 20). After a thorough review of the literature, no study has yet explored the psychological process by which increased social distancing due to patient care may escalate depression among nurses. Specifically, the mechanisms involving tenure, patient care, social distancing, and depression have not been examined. Therefore, we hypothesize that tenure, and work unit with acute care are associated with social distancing and depression. Moreover, the impact of loss of social support due to social distancing and the stigma related to caring for COVID-19 patients will increase the risk of depression. The main purpose of this study is to explore the correlation between depression, years of experience (tenure), working units and perceived stigma (social distance) on nursing staff. Additionally, this study examines if the mechanism of perceived social distancing as the mediator linking the work unit and tenure with depression although correlations between depression, social stigma of COVID-19 and demographics among healthcare professionals have been documented in literature.

## Research methods

### Design and participants

Research protocol has been reviewed and approved by the Research Ethics Committee of National Taiwan Normal University (Approval number 202003HS002). The study employed a cross-sectional survey among nurses in Taiwan in March 2020 using a respondent driven sampling technique. Anonymous and voluntary questionnaires were distributed via email, Facebook, LINE, and other online platforms to hospital nurses and request them to refer the link to their colleagues. Initial key informants were selected based on gender, age, and types of service institutions. A total of 1,123 nurses responded and data were collected. After excluding incomplete questionnaire, 1,064 questionnaires were obtained, resulting in a valid response rate of 94.75%.

### Measures

Demographics include age, gender, education level, years of clinical work (tenure), working unit, and exposure to COVID-19 patients. The hospital working unit is categorized into acute care units (such as isolation wards, internal medicine, emergency, and anesthesiology, abbreviated as ACC) and other non-ACC departments (such as pediatrics, family medicine, and radiology).

Perceived social distance as stigma. The social distancing scale during the COVID-19 outbreak was designed based on the Bogardus Social Distance Scale, which has been widely used in research related to mental illness and perceived discrimination (21). When respondents were asked whether they believe others would be willing to ride public transportation with them, shop with them, chat with them, shake hands with them, dine with them, kiss them, and whether others have displayed aggressive behaviors toward healthcare workers during the pandemic. Responses to these questions were rated on a five-point scale ranging from “impossible” to “very likely.” Scores were then summed across items, with an internal consistency reliability coefficient of 0.781 in this study.

Depression. This study utilized the 10-item version of the Center for Epidemiological Studies of Depression Scale (CES-D) (22). Each item in the scale is rated on a four-point Likert scale, and scores are summed across items. A score of 10 or higher indicates the presence of depression. A study examined psychometric properties of the CESD-R across a large community sample (*N* = 7,389) and showed good psychometric properties, including high internal consistency, strong factor loadings, and theoretically consistent convergent and divergent validity with anxiety, schizotypy, and positive and negative affect (23). The internal consistency reliability coefficient for the sample in this study was 0.85.

### Statistical analysis

The data were analyzed using SPSS statistical software version 22 and descriptive statistics was conducted to describe the data. In order to understand whether perceived social distancing during the early stages of a pandemic outbreak would affect their depression and whether there exists a mediating mechanism involving years of service, work unit, and depression, this study employed a structural equation model (SEM) with social distancing as the mediator for analysis. The rationale for using SEM is that it allows us to clearly specify our hypotheses and examine both direct and indirect relationships simultaneously between variables while accounting for measurement error.

## Results

As shown in Table 1, 1,064 nurses completed this study with 32 (3.01%) males and 1,032 (96.99%) females. With respect to age, 264 (24.8%) aged 20–29; 403 (37.9%) aged 30–39; and 397 (37.3%) aged 40 and over. Regarding the service unit, there were 535 (50.28%) nurses in acute care units and 529 (49.72%) in non-acute care units. The average tenure was 13.12 years. Among the 1,064 nurses, the perceived willingness of others to interact with nurses during the initial outbreak of COVID-19 was as follows: (1) willing to ride public transportation with nurses: 738 (74.5%). (2) Willing to go shopping with nurses: 818 (80.5%). (3) Willing to chat with nurses: 862 (83.9%). (4) Willing to shake hands with nurses: 480 (45.1%). (5) Willing to dine with nurses: 760 (74.4%). (6) Willing to kiss on the cheek: 437 (44.5%). (7) Others displaying aggressive behavior toward nurses during the pandemic: 305 (30.7%). The average social distance score was 4.78. The mean score of depression was 9.92 with 517 individuals scored 10 or higher on the CESD-10, accounting for 48.9%.

**Table 1:** Basic information of the nursing staff participating in this study.

Variable	*N*	%
Gander		
Male	32	3.01
Female	1,032	96.99
Age		
20–29	264	24.81
30–39	403	37.88
> = 40	397	37.31
Work unit		
Non-ACC	529	49.72
ACC	535	50.28

	Mean	SD
Tenure	13.12	7.80
Social distance	4.78	2.73
Depression	9.92	6.39

ACC (Acute Care Units) refers to high-risk acute care units such as internal medicine, emergency, and anesthesiology. Non-ACC units refer to units other than acute care units.

**Figure 1:**
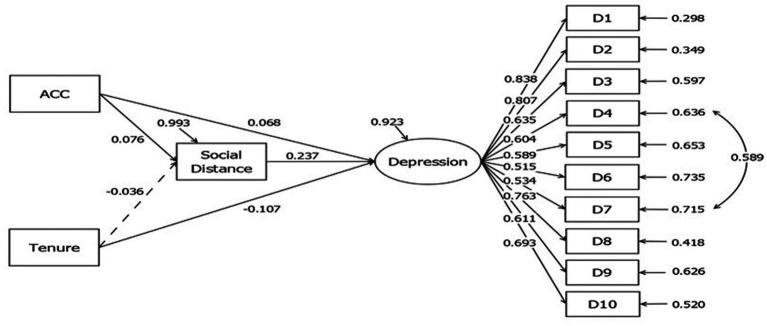
Structural equation model analysis with social distancing as the mediator variable for work unit, years of work experience, and depression. The model fit is good (Chi-square = 391.812, df = 61, chi-square/df = 6.42, RMSEA = 0.075, CFI = 0.928, SRMR = 0.045).

Social distancing was then employed as the mediator in the analysis of the relationship between work unit, tenure, and depression. As shown in Figure 1 and Table 2, results indicated that there is a positive correlation between work unit (ACC and non-ACC) and both social distancing and depression (*β* = 0.076 and 0.068, *p* < 0.01 respectively). There is no significant correlation between tenure and social distancing, but a negative correlation between tenure and depression was found (*β* = −0.107, *p* < 0.01), indicating that lesser work experience is associated with higher depression. Furthermore, there is a significant correlation between social distancing and depression (*β* = 0.237, *p* < 0.01). The direct effect of work unit on depression is 0.068 (*p* < 0.05). The indirect effect of work unit on depression through social distancing is 0.018 (*p* < 0.05). The total effect of tenure on depression is −0.115 (*p* < 0.01). The direct effect of social distancing on depression is 0.237 (*p* < 0.01).

**Table 2:** Standardized effects analysis of social distancing and depression.

	Social distance			Depression		
	Direct effect	Indirect effect	Total effect	Direct effect	Indirect effect	Total effect
Work unit^a^	0.076^*^	NA	0.076^*^	0.068^*^	0.018^*^	0.086^**^
Tenure	−0.036	NA	−0.036	−0.107^**^	−0.008	−0.115^**^
Social distance				0.237^***^		

^a^0 = Non-ACC, 1 = ACC. ^*^p < 0.05, ^**^p < 0.01, ^***^p < 0.001.

## Discussion and conclusion

This study explores how nurses perceive social distancing during the COVID-19 pandemic and how this perception potentially mediates the impact of their work environment and depression. The aim is to describe the depression on nurses during the COVID-19 and investigate how their work tenure and the workplace influence their awareness of social distancing and depression.

Our study found that substantial percentage of nurses experienced depression during the early era of COVID-19 pandemic and is consistent with prior studies. A study of 442 participants indicates that 51.6% (224) of healthcare workers experience anxiety, while 64.7% (286) experience depression, and 41.2% (182) stress (24). Lai et al. (25), in a survey of 1,257 healthcare workers caring for COVID-19 patients, found that risk factors for mental health problems included depression (50.4%), anxiety (44.6%), insomnia (34.0%), and distress (71.5%). Danilo and Robin (26) pointed out that prior to the outbreak of the COVID-19 pandemic, social distancing of such severity between individuals had never occurred before, and the social environment has a significant impact on people’s life satisfaction and happiness. Van Steenkiste et al. (27) conducted a prospective study analyzing the psychological health effects on frontline nursing staff caring for COVID-19 patients during hospitalization. Their study, which included a sample of 81 nurses, showed that nursing staff caring for COVID-19 patients during the pandemic experienced higher and more persistent distress scores, indicating a significant impact on their psychological health.

In this study, the finding that the work unit of nursing staff was significantly related to social distancing is not surprising. Nursing staff caring for infected patients in ACC units showed higher self-protection awareness compared to those in general units, and they were relatively more accepting of social distancing to protect others. The findings suggest that during the COVID-19 pandemic, the level of social distancing between individuals varies significantly depending on whether they work in ACC units or general units. Moreover, the self-protection awareness of nursing staff caring for infected patients also influences their behavior, further increasing social distancing.

However, working in ACC units is also correlated with higher level of depression although nurses knew the risk and keep social distancing. Elbay et al. (24) have found that the outbreak of the COVID-19 pandemic is a global catastrophe with significant impacts on the mental health of frontline healthcare workers. Nursing staff on the frontline of epidemic prevention face considerable psychological pressure when caring for confirmed/suspected infected patients, which should not be underestimated. Consistent with previous research findings, nurses caring for COVID-19 patients are more likely to experience psychological issues such as fear and discrimination, as well as feelings of exhaustion (28, 29). Another finding that a negative correlation between tenure and depression suggests that the longer the years of work experience, the lower the depression. Conversely, nursing staff with fewer years of work may experience higher levels of depression due to the impact of the COVID-19 pandemic. Based on the systematic review by Delassalle and Cavaciuti (20), there is a lack of effective intervention measures among nursing staff during COVID-19 regarding psychological distress. Yang et al. (30) conducted a systematic review and proposed that resilience has predictive power for psychological distress, their qualitative research highlighted the importance of policy and support provision in reducing psychological distress.

The finding that perceived social distance partially mediates between work units and depression. Nurses may worry about their young children or families at home or experience social discrimination due to their work (26, 31, 32). As previous studies have shown, despite having professional education and training, nursing staff still experience negative psychological reactions such as fear, compulsion, and sacrifice when caring for COVID-19 patients. They may even feel more exhausted or discriminated against compared to colleagues who do not directly care for confirmed patients (28, 29). The emergence of social distance may stem from the loneliness generated by interpersonal behaviors, and this negative feeling can directly or indirectly affect physical or psychological health (26). As healthcare supervisors, maintaining open communication channels, fostering positive interactions, providing robust support systems (including colleagues, family, friends, etc.), and ensuring a safe working environment with adequate protective equipment are essential. These measures are believed to significantly help nursing colleagues on the frontline caring for COVID-19 patients. For nursing staff working in ACC, it is crucial to provide emotional support and offer stress-relief channels. In addition to arranging relevant education and training, stress-relief courses, and providing open and transparent information on epidemic prevention, measures such as providing a safe and comfortable working environment and accommodation, implementing home support strategies, and organizing appropriate health promotion activities can be particularly beneficial for nursing staff directly caring for confirmed patients (14, 15). Furthermore, public education and awareness can also be conducted through government policies and social media platforms. By fostering understanding and empathy among the general public, society can provide maximum support to nursing staff working on the frontline of epidemic prevention, containment, and care for infected patients. This support enables these healthcare providers to fulfill their duties without worry, caring for patients in need of medical resources. Additionally, besides avoiding situations where nursing staff are stigmatized or marginalized, providing support for their physical, mental, and emotional well-being can effectively reduce their turnover intention.

This study has several strengths and weaknesses. First, the use of a relatively large sample size (1,064 participants) ensures more reliable analysis, although the web-based online survey may have selection biases, as it is uncertain whether all individuals were invited, and respondents were self-selected. Second, we employed a robust structural equation modeling (SEM) approach to investigate mediating relationships between work units, tenure, social distancing, and depression. However, the cross-sectional design limits our ability to draw causal conclusions. Third, we sent our invitation through various channels and hospitals, and fortunately, male nurses responded to our survey. Although the percentage of male nurses is small, this gender disparity may have affected our depression outcomes, as previous studies have suggested that female nurses are more likely to experience rumination and depression than male nurses (33, 34).

Lastly, it is also important to note that this study’s findings are limited to data collected via online surveys during the early stages of the pandemic. As the severity of the pandemic has gradually declined with the widespread availability of vaccines, the conclusions may not be easily extrapolated to the current status of all frontline nursing staff regarding their work experience, work units, social distance, and depression. COVID-19 is an emerging infectious disease with strong transmissibility. In addition to its potent infectiousness, the early stages of the pandemic resulted in numerous healthcare workers sacrificing their lives in the line of duty. Nursing staff are at the forefront of ethical and moral responsibilities, facing unprecedented psychological challenges and hardships, especially those working in negative pressure isolation wards (12).

Danilo and Robin (26) pointed out that during crises or disasters, widespread social isolation occurs, which can have significant impacts on life satisfaction and even life itself. During the initial outbreak of COVID-19 in Taiwan, when the transmission routes and vaccines were not yet clear, nursing staff who cared for suspected or confirmed infected patients not only avoided contact with their families but also faced ostracism from the public, causing them significant emotional distress. Several studies have focused solely on exploring the psychological stress experienced by nursing staff during the epidemic. However, this study, unable to engage in direct interpersonal contact at the time, utilized various online platforms to conduct surveys, providing a genuine depiction of the relationship between social distance and psychological depression among nursing staff in high-risk units. This approach yielded valuable insights into their experiences during the pandemic. Indeed, this study commenced during the early stages of the pandemic outbreak. With the widespread availability of vaccines and advancements in medical technology, the global trend of the pandemic has gradually cooled down. Consequently, it is challenging to further investigate the long-term psychological distress and related inferences regarding the impact of social distance on nursing staff in high-risk units. This limitation prevents a comprehensive understanding of the current situation and landscape of nursing staff at this stage.

Indeed, the COVID-19 pandemic has persisted globally for over 3 years. Unexpectedly, Taiwan experienced a shortage of nursing manpower in major hospitals after the COVID-19 lifting in 2023. In response, the government proposed several policies to address this nursing workforce issue, emphasizing the urgent need to improve the nursing practice environment (35). This study only focused on the correlation between stigma (social distancing) and depression among nursing staff during the early stages of the pandemic outbreak. Future research could continue exploring these correlations to promote a more supportive and friendly nursing work environment. Moreover, these findings can assist healthcare managers in developing more appropriate support systems and health promotion programs for frontline clinical healthcare workers. These results indicate that frontline nursing staff working in high-risk units and with shorter work experience face significant emotional challenges such as anxiety, depression, and stress. Therefore, healthcare supervisors can refer to guidelines provided by the Department of Nursing and Health Care, Ministry of Health and Welfare (36) to establish internal support mechanisms, conduct advocacy and education, provide psychological care services, and take action to support the physical and mental health of employees. For instance, providing free confidential counseling and mindfulness-based stress reduction, in addition to physical exercise, would benefit nurses in need.

## Data Availability

The raw data supporting the conclusions of this article will be made available upon reasonable request to the corresponding author.
